# The Evolution of Radiation Therapy in Metastatic Breast Cancer: From Local Therapy to Systemic Agent

**DOI:** 10.1155/2018/4786819

**Published:** 2018-05-16

**Authors:** Jessica M. S. Jutzy, Jeffrey M. Lemons, Jason J. Luke, Steven J. Chmura

**Affiliations:** ^1^Department of Radiation Oncology, University of Chicago, Chicago, IL, USA; ^2^Department of Medicine, University of Chicago, Chicago, IL, USA

## Abstract

Radiation therapy is a mainstay of treatment in early and locally advanced breast cancer but is typically reserved for palliation of symptomatic lesions in patients with metastatic breast cancer. With new advances in the field of tumor biology and immunology, the role of radiation in the metastatic setting is evolving to harness its immune-enhancing properties. Through the release of tumor antigens, tumor DNA, and cytokines into the tumor microenvironment, radiation augments the antitumoral immune response to affect both the targeted lesion and distant sites of metastatic disease. The use of immunotherapeutics to promote antitumoral immunity has resulted in improved treatment responses in patients with metastatic disease and the combination of radiation therapy and immunotherapy has become an area of intense investigation. In this article, we will review the emerging role of radiation in the treatment of metastatic disease and discuss the current state of the science and clinical trials investigating the combination of radiation and immunotherapy.

## 1. Introduction

Radiation therapy is traditionally utilized in stages I–III breast cancer as a local therapy after surgical management to improve disease-free survival and in some cases overall survival. In the metastatic setting, it is used for effective palliation of symptomatic metastases. Advances in tumor biology and immunology have led some to suggest a role for radiotherapy in the metastatic setting to augment traditional systemic therapies such as chemotherapy or immune-modulating agents. While decades of research have demonstrated that a major component of local tumor control is mediated by irreparable damage to the DNA of malignant cells resulting in cell death [1], recent research has elucidated multiple radiation-induced effects on both tumor cells and the tumor microenvironment. Following ablative doses of radiotherapy, release of tumor antigens, tumor DNA, cytokines, and chemokines promote innate intratumoral immunity, leading in some cases to an adaptive response [2, 3]. This suggests that the immune response may play a part in high local control rates seen with radiation therapy. Immune-modulating therapeutics, such as checkpoint inhibitors, might therefore be incorporated with radiation to enhance the antitumor immune response with the intent of improving outcomes in patients with oligometastatic or polymetastatic disease [2]. Here we will review the rationale for the use of stereotactic body radiotherapy (SBRT) for the treatment of oligometastatic breast cancer and explore the data to suggest that incorporating immunotherapy may expand the use of SBRT to polymetastatic disease.

## 2. Oligometastases in Breast Cancer

Breast cancer has provided one of the earliest models in our understanding of cancer progression and metastasis. Pioneering work by Halsted resulted in the theory of orderly spread of cancer from the initial primary tumor location to the regional draining lymph nodes, followed by metastatic spread to distant organs [4]. When subsequent radical surgical interventions and en bloc resections did not eliminate the occurrence of distant metastases, competing hypotheses by Keynes and subsequently Fisher proposed that breast cancer was likely already systemically disseminated at the time of diagnosis at the primary tumor [5, 6]. With this came a shift from aggressive surgery to neoadjuvant and adjuvant systemic therapies, with the goal of targeting disseminated microscopic disease.

A third hypothesis established by Hellman, the “spectrum theory,” stated that breast cancer more likely existed as a spectrum of localized and widespread disease [7]. Indeed, in clinical practice, patients present within a spectrum of involvement, some with localized primary tumors, and others with disseminated metastases at time of initial presentation. Hellman and Weichselbaum later refined this theory with the description of an intermediate disease state between localized and widely metastatic cancer [8]. This disease state, known as “oligometastases,” was defined as a clinical state of limited metastases, typically one to five lesions, in a single or limited number of distant sites.

The concept of oligometastases has resulted in a change in therapeutic goals for women with metastatic breast cancer and a limited burden of disease, as these patients may be able to achieve durable disease control with ablation of their metastases. In recently published first-line metastatic breast cancer trials, many patients were found to have fewer than 4 sites of disease and those with fewer metastases had improved outcomes. Similar findings have also been noted in large cohort studies of breast cancer patients, in which more than 25% of early-stage breast cancer patients had 1–5 metastases at time of disease progression [9]. These women were noted to have improved median survival compared to those with >5 metastases (108 versus 22 months). A meta-analysis of metastatic breast cancer patients who received anthracycline-based systemic therapy showed that those with 1–5 metastases had significantly improved outcomes compared to those with >5 metastases [10]. These studies demonstrate that a clinically significant population of oligometastatic breast cancer patients exists and may achieve long-term disease control with aggressive treatment of their metastatic disease and form the rationale for continued clinical investigations [2].

## 3. Ablative Treatment of Oligometastatic Breast Cancer

Standard of care for metastatic breast cancer includes systemic therapy in the form of hormonal, biologic, and cytotoxic agents. Ablative treatment for metastases using radiation or surgery is typically reserved for palliation of symptomatic lesions. Even in patients with good performance status and limited metastatic disease, few achieve complete and durable responses with systemic agents alone. In a study of over 1,500 women treated with doxorubicin and alkylating chemotherapy for metastatic breast cancer, only 1.6% achieved complete response and were free of relapse at 15 years [11]. Interestingly, 8 of those patients who achieved lasting response received metastasis-directed therapy with radiation, surgery, or chemoembolization, suggesting that local therapies could improve outcomes in selected patients.

The idea that certain patients with limited metastatic disease may derive lasting benefit from more aggressive treatment of their metastatic disease has resulted in multiple retrospective and single-arm studies evaluating outcomes with local therapy and/or metastasectomy. Several studies in various cancer types have shown that standard systemic therapy combined with the elimination of all clinically detected metastases via surgery or ablative radiotherapy results in superior disease control than systemic therapy alone [12–14]. Surgical resection of metastases in breast cancer patients has shown promising results with regard to progression-free survival and overall survival [14]. Pockaj et al. demonstrated favorable median progression-free survival (14–34 months) and overall survival (24–63 months) after surgical resection of liver metastases [15].

In metastatic breast cancer patients who are poor surgical candidates due to medical comorbidity or metastases that are unresectable due to location or invasion, radiotherapy can be utilized for the treatment of limited metastatic sites. A study of patients treated with ablative radiation therapy for limited metastatic disease on two sequential protocols from University of Rochester demonstrated an 89% local control of treated metastases, with 4-year actuarial overall survival of 59% and progression-free survival of 38% in those with metastatic breast cancer [16]. Multivariate analysis revealed that patients with primary breast cancer, single bony metastases, and stable or responsive disease prior to SBRT had the largest benefit from ablative radiation therapy. However, due to the lack of published randomized prospective trials evaluating the aggressive treatment of oligometastatic disease, it is uncertain whether these reported series pointing to a long-term survival after local therapeutic interventions in breast cancer patients with limited metastatic disease are generalizable. In carefully selected patients, aggressive local therapy may be beneficial. The mechanism behind this benefit remains unclear but may result from inhibiting progression of metastatic disease, preventing additional metastatic seeding, or through an immunostimulatory abscopal effect on micrometastatic disease.

## 4. Immunooncology and Enhancing Abscopal and Adscopal Effects

The immune system is a powerful network of cells providing surveillance to prevent the development and progression of malignancy. Avoiding immune destruction is critical in cancer progression and is an important hallmark of cancer pathogenesis [17]. Enhancing the immune system's ability to detect and destroy cancer cells and inhibiting immune escape mechanisms, both key elements of immunooncology (IO) therapy, represents a transformational approach to cancer care with a potential for long-term antitumor responses.

The effector T cell response to cancer is controlled by a balance of antigen presentation to the T cell receptor (TCR) and subsequent costimulatory and coinhibitory signals [18]. Initially, immunotherapies were developed to target negative regulators of the T cell receptor response, primarily cytotoxic T-lymphocyte associated protein 4 (CTLA-4) and programmed cell death protein 1 (PD-1). Inhibition of these negative regulatory receptors, referred to as immune checkpoint blockade, results in the enhanced activation of T cells and potent antitumor activity.

Early studies in mouse models of metastatic breast cancer, radiotherapy combined with immune-mediated inhibition of CTLA-4 demonstrated a significant survival advantage compared to groups treated with radiotherapy alone [19]. Subsequent human studies with CTLA-4 blockade have demonstrated improved overall survival in patients with metastatic melanoma and studies using anti-PD-1 antibodies such as pembrolizumab, nivolumab, and avelumab have shown disease response in patients with non-small cell lung cancer, melanoma, breast, and renal cell carcinoma [20–23]. The success of these agents has resulted in a multitude of new immunotherapy agents, which are now being investigated in an attempt to harness the natural immune response against malignant cells. In addition to enhancing the T cell response directly, further agents are being developed to target cell populations beyond effector T cells such as natural killer (NK) cells, T regulatory (Treg) cells, tumor-associated macrophages (TAMs), and dendritic cells (DCs).

Several of the key activation steps required for effective systemic immunotherapy are also impacted by radiation [2, 18]. As with any immune reaction, the initial response is triggered via innate immunity identifying the presence of an infection or tumor, leading to antigen capture/processing and presentation to the adaptive immune compartment, classically including T cells. Tumor cell death induced by radiation can result in DC cross-priming and subsequent activation of the stimulator of interferon genes (STING) pathway [1], a major driver of type I interferons [1, 23, 24]. This type I interferon (IFN) release acts as a bridge between innate and adaptive immunity, improving DC activation and recruitment to the tumor as well as inducing type II interferon and leading to cytotoxic T cell (CTL) activation [2, 25, 26]. In mouse models, induction of type I IFN by radiotherapy activates proapoptotic signaling cascades within the tumor cells, while also increasing activation of antigen-presenting cells (APCs) and T cells [27–29]. Additionally, this radiation-induced IFN secretion has been shown to increase the release of chemokines CXCL10 and CXCL16, which enhances cytotoxic and type 1 helper T cell migration into the tumor and increases cytotoxic and helper T cells' killing capability via upregulation of Fas/FasL [2, 3, 30].

Although radiation has been shown to have immunostimulatory effects, immunosuppressive effects can also be observed with IFN-*γ* associated recruitment of regulatory T cells (Tregs) and myeloid derived suppressor cells (MDSCs) [2, 18]. The increase in Treg cells in the tumor microenvironment results in increased IL-10 and TGF-beta production that stimulates MDSCs, which results in suppression of T cell activation and the promotion of tumor regrowth. Additionally, TGF-beta has been shown to inhibit T cell activation. Mouse models of breast carcinoma treated with TGF-beta blocking agents show enhanced priming of CD8+ T cells to endogenous tumor antigens released by radiation [31]. Low-dose radiation can also increase recruitment of tumor-promoting M2 type TAMs [32]. Arginase, an enzyme overexpressed in M2 type TAMs, results in depletion of arginine, which impairs expression of the T cell receptor (TCR) zeta chain that is necessary for T cell activation and proliferation [33, 34]. Preclinical studies of inflammatory breast cancer have demonstrated enhancement of angiogenesis, metastasis, and invasiveness due to IL-6 secretion by M2 TAMs, resulting in accelerated tumor growth [35].

By combining immunotherapy agents with radiation, the balance of these immunostimulatory and immunosuppressive effects could be shifted to improve local and possibly distant disease control in oligometastatic patients. Indeed, this effect was shown in mouse models, where concurrent administration of anti-PD-1 and anti-PD-L1 antibodies with fractionated radiotherapy generated CD8+ T cell responses that improved local tumor control [35].

A phenomenon known as the abscopal effect has been described in some patients with metastatic disease who received treatment with radiation therapy to one or more metastatic lesions and had nonirradiated tumors shrinking outside the radiation field. This effect was first described by Mole in the 1950s and is thought to be an immune-mediated tumor response [36]. Abscopal effects in untreated lesions have been demonstrated in mouse tumor models treated with radiation and immunotherapy, with augmented T cell responses in both primary and metastatic lesions [37, 38]. Radiation has been shown to increase PD-L1 expression on tumor cells, resulting in an impaired T cell response. The addition of anti-PD-L1 therapy after radiation in these studies resulted in a synergistic amplification of tumor killing mediated by TNF-alpha signaling, increased CD8+ T cells, and decreased MDSC within tumors [38]. In the clinical setting, metastatic melanoma patients being treated with ipilimumab who received palliative radiation with 28.5 Gy in three fractions had regression of the radiated lesion as well as other areas of metastatic disease [39]. One patient with metastatic NSCLC treated with ipilimumab and radiation not only showed a dramatic initial response in the treated and untreated sites of disease but also had a durable response with no evidence of disease 1 year later [40]. In addition to effects on distant lesions, local or “adscopal” responses within the irradiated target or an unirradiated portion of the contiguous tumor have been described in patients treated with radiation and PD-1 blockade [41, 42].

Several new immunotherapeutics show promise for combination with radiation due to their parallel effects on immune cell function to amplify these abscopal and adscopal effects. Nivolumab and pembrolizumab are antibodies that target the negative regulatory molecule programmed death-1 receptor (PD-1; CD279). PD-1, a cell surface membrane receptor expressed by activated T and B lymphocytes, functions to downregulate lymphocyte activation upon interaction with its ligands PD-L1 and PD-L2. Inhibition of this interaction promotes antigen-specific T cell responses to both foreign and self-antigens as well as increased IFN-*γ* associated gene expression. As radiation induces T cell migration into the tumor, the addition of a checkpoint inhibitor may further enhance T cell-mediated killing of the tumor. The phase Ib KEYNOTE-012 pembrolizumab study evaluated 111 heavily pretreated patients with advanced triple negative breast cancer and showed an 18.5% response rate [43] and the results of a single-agent phase II study of pembrolizumab in advanced triple negative breast cancer were recently reported. Six-month progression-free survival was 12% and overall survival was 69% in the 170 patients enrolled [44]. Similar results have been seen with PD-L1 inhibitors atezolizumab and avelumab. The results of a phase II study of atezolizumab showed overall response rates (ORR) of 26% in first-line patients and 7% in second-line (2L+) patients [45]. Overall survival was 41% at 1 year and 22% at 3 years. A phase III trial is now underway in triple negative breast cancer of atezolizumab with or without paclitaxel [46]. In the phase Ib JAVELIN solid tumor study, 168 patients with metastatic breast cancer showed a 16.7% ORR in PD-L1+ tumors [47]. Combining these agents with radiation could amplify the response rate seen with pembrolizumab alone by increasing activated T cell migration to the tumor through the radiation-induced release of chemokines.

Ipilimumab is a human monoclonal IgG1*κ* antibody against human CTLA-4 (CD152), a negative regulator of T cell activity expressed on a subset of activated T cells. Ipilimumab blocks the interaction of CTLA-4 and CD80/86, which allows for increased T cell activation and clonal expansion via an independent mechanism from PD-1 blockade [48]. Additionally, CTLA-4 blockade reduces Treg function, thereby preventing Treg-mediated inhibition of T cell activation and cytotoxic killing. Ipilimumab has shown a statistically significant survival benefit in advanced and metastatic melanoma in phase III trials [49]. In breast cancer, a pilot study evaluating tumor-infiltrating lymphocyte (TIL) profiles in 19 early-stage breast cancer patients treated with preoperatively with ipilimumab alone and in combination with cryoablation demonstrated increased intratumoral T cell density [50]. The ability of ipilimumab to decrease Treg function and increase T cell infiltration would increase the effectiveness of radiation therapy by reducing the immunosuppressive effects of radiation while simultaneously increasing the stimulatory power.

In the same vein as checkpoint inhibitors, 4-1BB agonists promote T cell activation. As members of the TNFR-TNFL family, 4-1BB (CD137) and 4-1BBL provide a costimulatory signal to CD4+ and CD8+ T cells which upregulate antiapoptotic genes to promote T cell survival. In mouse models of metastatic breast cancer, treatment with 4-1BB agonists resulted in a regression of metastatic tumors and increased survival, which was further increased with the addition of radiation [51–53]. As the 4-1BB signal provides a positive costimulatory signal, combination with blockade of the PD-1/PD-L1 negative costimulatory signal could further enhance the radiation-induced antitumoral T cell response.

LAG-3 (CD223) is a type 1 transmembrane protein that promotes Treg activity and is required for the maximal suppression of T cell activation and proliferation [54]. High LAG-3 expression has been shown on exhausted CTLs, which results in limited antitumor CTL responses [55, 56]. A phase I study in ER+ metastatic breast cancer patients using a combination of a LAG-3 antagonist with paclitaxel showed an improved objective response rate of 50% compared to a historical rate of 25% with paclitaxel alone and a phase IIb study is underway to further investigate its effect in this patient population [57]. It is possible that this impressive response rate could be further improved in combination with SBRT to increase antigen presentation and enhance the antitumor immune response.

Cabiralizumab is a recombinant, humanized IgG4 monoclonal antibody that binds to the human colony-stimulating factor 1 receptor (CSF1R) and prevents binding of its ligands' colony-stimulating factor 1 (CSF1) and interleukin-34 (IL-34). CSF1 is required for survival of immunosuppressive TAMs that promote angiogenesis and tumor metastasis and studies in mouse models of breast cancer have shown TAM recruitment into the tumor due to CSF-1/CSF-1R signaling [58, 59]. Subsequent blocking of CSF-1 signaling results in reduced TAM migration and improved response to subsequent therapies [60]. Preclinical studies in inflammatory breast cancer demonstrate significant reduction in M2 TAM populations in mice treated with anti-CSF1 antibodies [61]. The reduction in TAM migration into the tumors resulted in decreased IL-6 signaling and delayed tumor growth and skin invasion. Using CSF1R inhibitors to effectively deplete the TAM population in breast cancer patients could allow for improved response of metastatic lesions subsequently treated with SBRT or fractionated radiation.

## 5. Harnessing the Abscopal and Adscopal Effects with SBRT and Immunotherapy

Mounting evidence suggests that, apart from its direct effects, radiotherapy, and particularly SBRT, can act as a trigger for the innate and adaptive immune system. However, despite these effects, many patients with limited metastatic disease treated with radiation alone will frequently experience disease progression leading to widely metastatic disease. Although radiation can induce activation of T cells and tumor infiltration by DCs and antigen-presenting cells, the accompanying Treg cells, MDSCs, and TAMs prevent a more robust immune response. The combination of radiotherapy with immune-modulating agents could enhance the effectiveness of radiation in treating oligometastatic breast cancer by shifting the balance away from the immunosuppressive effects using anti-LAG3 and anti-CSF1R agents and simultaneously enhancing the immunostimulatory effects via checkpoint inhibition with anti-PD-1/PD-L1 and increased T cell survival with 4-1BB agonists. Although radiotherapy alone does not provide a sufficient antitumor immune response to achieve a clinically meaningful effect at untreated sites, augmenting the immune response with the addition of immunotherapy to SBRT may increase the downstream effects of radiation-induced IFN release and induce abscopal and adscopal effects more frequently [62].

Many questions remain regarding the best method for combining radiation and immunotherapy. Currently, the most effective radiation dose fractionation for this combined modality treatment is not known. The available data on fractionation and its effect on the radiation-induced immune response are conflicting. Animal models combining anti-CTLA-4 agents with radiation showed an improved abscopal effect with 8 Gy × 3 dosing over a single ablative dose of 20 Gy [63]. Evaluation of TGF-beta blockade in concert with radiation in a mouse breast cancer model utilized 6 Gy × 5 and demonstrated enhanced T cell infiltration into radiated tumors [31]. Mouse studies evaluating MDSC and T cell infiltration showed increased T cell infiltration and lower MDSC numbers with a single 30 Gy dose compared to 3 Gy × 10 dosing [64]. Although optimal fractionation has not been determined, the ability of SBRT to target and treat metastatic lesions that are not amenable to surgery in a short time makes it an attractive option for combination with immunotherapy.

In addition to uncertainty of fractionation, there is also concern regarding the safety of combining immunotherapeutics with radiation. Multiple phase I and II studies are ongoing to investigate the safety of this approach, primarily with the use of single-fraction or hypofractionated radiation in conjunction with checkpoint inhibitors [2, 41, 64–66]. The University of Chicago recently published the results of a phase I trial of 73 participants combining multisite SBRT as per BR-001 followed by anti-PD-1 therapy and thus far has not observed any synergistic toxicity relative to either approach alone [41]. Objective overall response rate was 13.2% for all patients. Memorial Sloan Kettering and Cedars Sinai have completed a phase II trial investigating palliative radiation and pembrolizumab in metastatic triple negative breast cancer. Patients were treated with 30 Gy delivered in five fractions with pembrolizumab administered within three days of the first radiation treatment. Of 17 patients enrolled in the study, 3 had durable partial responses both in field and out of field, with 60%, 54%, and 34% decrease in tumor burden by RECIST, with no significant toxicities observed [65]. For patients with brain metastases, both stereotactic radiosurgery (SRS) and whole-brain radiotherapy with conventional fractionation which were investigated with tremelimumab were well tolerated, and 2 of 6 women with Her2+ disease had durable responses [66]. Two phase I studies at Weill Cornell Medical College are investigating anti-TGF-beta therapeutics in combination with radiation to induce abscopal responses. The first utilized an anti-TGF-beta antibody, fresolimumab, at two doses and radiation of 22.5 Gy in 3 fractions [67]. The higher fresolimumab dose was associated with high median overall survival compared to the low-dose arm, and seven grade 3 or 4 adverse events were noted out of 23 patients in the study. The second trial, which is currently enrolling, evaluates a small molecule inhibitor of TGF-beta, galunisertib, in combination with radiation (NCT02538471).

As the number of clinical trials investigating immunotherapy and radiation rapidly increases, several questions for optimal trial design are still unanswered. Radiation dosing and fractionation are widely varied between ongoing studies, although most are using hypofractionated or single-fraction regimens. Additionally, the ideal treatment field size is unknown. The out-of-field adscopal effects seen in partially irradiated tumors in the study by University of Chicago bring up the possibility that, for large tumors, coverage of the entire lesion may not be required [41]. While the ongoing trials will provide useful information regarding the safety of combining radiation and immunotherapy, additional trials to evaluate optimal sequencing, radiation dosing, and field size are needed to further advance this combination therapy.

## 6. Conclusions

Currently, there is evidence that radiation can alter the immune profile of the tumor microenvironment by increasing tumor-antigen presentation, altering cytokine and chemokine release, and recruiting T cells to the tumor, with both antitumor and protumor effects. The promising results in initial immunotherapy trials in metastatic breast cancer and animal data showing enhanced tumor killing and abscopal and adscopal effects when immunotherapeutics are combined with radiation provide an interesting hypothesis that enhancing the immune response could improve patient outcomes and overall survival. However, the science behind radioimmunotherapy is based primarily on data from animal models and has not been fully evaluated in patients. Further study of both the safety and effectiveness is needed before this combined modality therapy is widely implemented.
